# Editorial: Women in experimental pharmacology and drug discovery: 2021

**DOI:** 10.3389/fphar.2022.1077670

**Published:** 2022-11-09

**Authors:** Tea Lanišnik Rižner, Csilla Özvegy-Laczka

**Affiliations:** ^1^ Faculty of Medicine, Institute of Biochemistry and Molecular Genetics, University of Ljubljana, Ljubljana, Slovenia; ^2^ Drug Resistance Research Group, Institute of Enzymology, Research Centre for Natural Sciences, Budapest, Hungary

**Keywords:** chemerin, osteoprotegerin, osteoporosis, Nrf2, premature ovarian failure, SLCO4A1, high-grade serous ovarian cancer, SLCO3A1_V3

Women are still underrepresented in research worldwide. Thus, it is crucial to encourage young women to decide for a career in science. There are many obstacles on the scientific path of a woman, and many women still describe reaching the glass ceiling whilst attempting to follow their aspirations. As female researchers working in the fields of pharmacology/biochemistry and as principal investigators leading groups that include mainly women, we have gathered experience in all aspects of research life. We thus gladly accepted the invitation to serve as guest editors of a Research Topic dedicated to promoting research by female scientists across all fields of Experimental Pharmacology and Drug Discovery, with an emphasis on submissions with women as first and/or corresponding authors.

This Research Topic includes contributions from four young female researchers from China, Pakistan, Austria, and Hungary. Their research highlights the diverse fields of Pharmacology. By chance, the submitted research mainly focuses on molecular aspects of conditions and pathologies that mainly affect women in their reproductive and post-reproductive periods. These include premature ovarian failure (POF), osteoporosis, and high-grade serous ovarian cancer (HGSOC), but also more general aspects regarding transporters responsible for the uptake of steroid precursors, prostaglandins, vasopressin, thyroxine, and different drugs in the brain and testes.

Zhang et al. investigated the role of oxidative stress and possible protective signaling pathways in POF. POF affects 1–3% of women, and its risk factors are heterogeneous and include oxidative damage. Currently, the only available chemical treatment for these patients is hormone therapy; however, this can increase the risk of gynecological cancers and other diseases. Zhang et al. studied the role of nuclear factor erythroid-2 related factor 2 (Nrf2), a transcription factor that regulates cellular responses to oxidative stress in a chemically induced POF mouse model. They demonstrated that Nrf2 knock-out mice are more susceptible to POF. They also showed that daphnetin, a Chinese herbal medicine and known activator of NRF2 signaling, plays a protective role in this chemically induced POF model. Furthermore, they deciphered the participants in daphnetin-Nrf2 signaling pathways. With this, the authors have demonstrated a potential alternative milder treatment for POF, compared to hormone replacement therapy.

Tariq et al. investigated the effects of the current treatment of osteoporosis by bisphosphonates on circulating adipokine levels. Osteoporosis is the most common chronic metabolic bone disease and is characterized by decreased bone mineral density. Previous studies revealed that adipokines have various effects on bone metabolism and remodeling. Tariq et al. determined blood levels of chemerin, vaspin, omentin-1, and osteoprotegerin (OPG) in healthy postmenopausal women and osteoporosis patients before and after therapy with Ibandronate (a bisphosphonate drug). They found higher chemerin and lower OPG levels in osteoporosis patients compared to healthy women. After treatment, chemerin levels decreased, whereas OPG and vaspin levels increased. Additionally, their multivariate linear stepwise regression analysis identified serum chemerin and OPG as independent predictors of bone mineral density. These adipokines may contribute to the list of blood biomarkers for diagnosing osteoporosis.

Solute carrier (SLC) transporters are being increasingly recognized in health and disease, as also indicated by two papers submitted to this special issue. Organic anion-transporting polypeptides (OATPs) from the SLC superfamily are exchangers of organic solutes residing (mostly) in the plasma membrane of epithelial or endothelial cells. They mediate the uptake of steroid hormone precursors and hence have been identified as potential targets for treating hormone-dependent cancers. Koller et al. investigated the expression of *SLCO4A1*, which encodes the steroid and prostaglandin transporter OATP4A1, in 33 HGSOC patient-derived cell lines. HGSOC has poor prognosis because it is often diagnosed at later stages, when tumor metastases are already present. These patient-derived cell lines are diverse and represent a valuable tool to better model *in vivo* conditions than generally available cell lines. *SLCO4A1* was variably expressed in these cell lines and HGSOC tissue samples. Further investigations of previously published data revealed a positive correlation between OATP4A1 and the ATP-binding cassette transporter ABCC3. Furthermore, high *SLCO4A1* expression was connected to inflammation-associated pathways, and low *SLCO4A1* expression to mitochondrial dysfunction but not estrogen-associated pathways. Based on these results, the major role of OATP4A1 in HGSOC may involve prostaglandin E2 uptake.

Besides their role in the cellular uptake of endobiotics, several members of the OATP family also transport drugs and can influence drug absorption, distribution, and elimination. These drug transporters, including OATP1A2, OATP1B1, OATP1B3, and OATP2B1, have been extensively investigated. However, other members of the OATP family are significantly less well-studied. One such member is OATP3A1, which is most abundantly expressed in the human brain and testes, where it can potentially mediate steroid hormone uptake. Bakos et al. cloned a novel functional isoform of OATP3A1 (OATP3A1_V3) and demonstrated that it shared substrates with the other two previously known OATP3A1 isoforms (OATP3A1_V1 and OATP3A1_V2). However, the novel variant localizes to the opposite (apical) membrane of the choroid plexus than OATP3A1_V1. The distinct localization of OATP3A1 isoforms in the choroid plexus may ensure the movement of steroid hormones from the blood to the central nervous system.

The molecular bases of POF, osteoporosis, and HGSOC and pharmacological actions of herbal medicines (e.g., Daphnetin (7,8-dihydroxycoumarin)) are still not completely understood. Furthermore, the effects of bisphosphonates on adipokines and the (patho)physiological roles of individual OATP transporters and their splice variants deserve clarification. This Research Topic comprising four articles by young female researchers provides new knowledge and thus fills certain gaps in the fields of Experimental Pharmacology and Drug Discovery but also highlights the importance of further studies.

